# The Cannabinoid Receptor *Type 2* as Mediator of Mesenchymal Stromal Cell Immunosuppressive Properties

**DOI:** 10.1371/journal.pone.0080022

**Published:** 2013-11-27

**Authors:** Francesca Rossi, Maria Ester Bernardo, Giulia Bellini, Livio Luongo, Antonella Conforti, Iolanda Manzo, Francesca Guida, Luigia Cristino, Roberta Imperatore, Stefania Petrosino, Bruno Nobili, Vincenzo Di Marzo, Franco Locatelli, Sabatino Maione

**Affiliations:** 1 Department of Women, Child and General and Specialistic Surgery, Second University of Naples, Naples, Italy; 2 Department of Onco-Haematology, IRCCS “Bambino Gesù” Children Hospital, Rome, Italy; 3 Department of Experimental Medicine, Second University of Naples, Naples, Italy; 4 The Endocannabinoid Research Group (ERG) via Campi Flegrei 34, Pozzuoli, Naples, Italy; 5 Institute of Cybernetics, National Research Council (CNR), Pozzuoli, Naples, Italy; 6 Institute of Biomolecular Chemistry, National Research Council (CNR), Pozzuoli, Naples, Italy; 7 University of Pavia, Pavia, Italy; Sudbury Regional Hospital, Canada

## Abstract

Mesenchymal stromal cells are non-hematopoietic, multipotent progenitor cells producing cytokines, chemokines, and extracellular matrix proteins that support hematopoietic stem cell survival and engraftment, influence immune effector cell development, maturation, and function, and inhibit alloreactive T-cell responses. The immunosuppressive properties of human mesenchymal stromal cells have attracted much attention from immunologists, stem cell biologists and clinicians.

Recently, the presence of the endocannabinoid system in hematopoietic and neural stem cells has been demonstrated. Endocannabinoids, mainly acting through the cannabinoid receptor *subtype 2*, are able to modulate cytokine release and to act as immunosuppressant when added to activated T lymphocytes.

In the present study, we have investigated, through a multidisciplinary approach, the involvement of the endocannabinoids in migration, viability and cytokine release of human mesenchymal stromal cells.

We show, for the first time, that cultures of human mesenchymal stromal cells express all of the components of the endocannabinoid system, suggesting a potential role for the cannabinoid CB2 receptor as a mediator of anti-inflammatory properties of human mesenchymal stromal cells, as well as of their survival pathways and their capability to home and migrate towards endocannabinoid sources.

## Introduction

Mesenchymal stromal cells (MSCs) are non-hematopoietic, multipotent progenitor cells able to differentiate into bone marrow (BM) stroma, as well as into adipocytes, chondrocytes, and osteocytes.

Initially expanded from BM, MSCs can also be culture-expanded from other sources, including umbilical cord blood, adipose tissue, and dental pulp [1], [2].

Because a specific marker for human MSCs has not been identified, the International Society for Cellular Therapy has recommended the following minimum criteria for defining multi-potent human MSCs (hMSCs) [3], [4]: (i) adherence to plastic under standard culture conditions; (ii) positive expression of CD105, CD73 and CD90 and negative expression of the hematopoietic cell surface markers CD34, CD45, CD11a, CD19 or CD79a, CD14 or CD11b and HLA-DR; and (iii) following specific stimulation, differentiation into osteocytes, adipocytes and chondrocytes *in vitro*.

MSCs produce cytokines, chemokines, and extracellular matrix proteins that support hematopoietic stem cell (HSC) survival and engraftment, influence immune effector cell development, maturation, and function, and inhibit alloreactive T-cell responses [5], [6].

The immunosuppressive properties of hMSCs have attracted much attention from immunologists, stem cell biologists and clinicians. Indeed, MSCs modulate the immune response by their interaction with a wide range of immune cells, including T cells, B cells, dendritic cells (DCs), regulatory T cells (T regs), natural killer (NK) cells, and NK T cells. The inhibitory function of MSCs is dependent on cell–cell contact and release of soluble factors, such as interleukin-6 (IL-6), IL-10, indoleamine-deoxygenase (IDO), transforming-growth factor β (TGF-β), interferon-γ (INF-γ), prostaglandin E2 (PGE2), and nitric oxide (NO) [7], [8].

MSC immunoregulatory ability is independent of the major histocompatibility complex. Based on this evidence, administration of MSCs has been suggested as an alternative strategy for treating immune-mediated diseases, and their immunosuppressive properties have been explored in a number of experimental autoimmune diseases, as well as in organ transplantation [9]–[12].

There is evidence demonstrating alteration in cytokine release induced by endocannabinoids on immune cells and that endocannabinoids such as anandamide (AEA) and 2-arachidonoylglycerol (2-AG) can be immunosuppressive when added to activated T lymphocytes, acting mainly through the endocannabinoid subtype 2 (CB2) receptor [13]. Contrary to the cannabinoid receptor *subtype 1* (CB1) receptor, which is particularly abundant in the central nervous system (CNS), the CB2 receptor is mainly expressed in spleen, tonsils, and immune cells, but has been found also in osteoclasts, in microglia and in keratinocytes, and skin tumor cells [14]–[17].

The CB2 receptor is involved in immune regulation by suppressing immune cell activation, through modulation of T-helper cell types 1 and 2 (Th1 and Th2) [18], inhibition of pro-inflammatory cytokine production [19], and nuclear factor-B-dependent apoptosis [20]. Indeed, a CB2 functional variant has been associated with several inflammatory/immune-based diseases [21]–[24]. Moreover, recent studies highlighted the presence of the endocannabinoid system in hematopoietic and neural precursor stem cells [25]–[28].

In the present study, we have investigated the involvement of endocannabinoids in hMSC migration, viability and cytokine release. Specifically, we observed up-regulation of the CB2 receptor expression (together with CB1-receptor down-regulation) during hMSC maturation and, following selective CB2 stimulation, the activation of the PI3K-AKT-mTOR pathway, possibly promoting cell survival and protein synthesis and/or increased metabolism.

## Materials and Methods

### Source of MSCs

The study was approved by the Second University of Naples Ethical Committee and written informed consent was obtained from donors. All clinical investigation have been conducted according to the principles expressed in the Declaration of Helsinki. We used MSCs isolated from residual cells of 12 healthy subjects (8 males, 4 females, median age 36.5±16 years, range 20–53), who donated BM for transplantation at the Bambino Gesù Children's Hospital.

### hMSC cultures

Mononuclear cells were isolated from BM by density gradient centrifugation (Ficoll 1.077 g/ml; Lympholyte, Cedarlane Laboratories Ltd., The Netherlands) and plated in non-coated 75–175 cm^2^ polystyrene culture flasks (Corning Costar, Celbio, Milan, Italy) at a density of 160,000/cm^2^. The complete culture medium consisted of LG-DMEM (Euroclone, Milan, Italy) supplemented with 10% fetal bovine serum (FBS; Gibco, Life Technologies Ltd, Paisley, UK), penicillin 50 U/ml, 50 mg/ml streptomycin and 2 mM L-glutamine (Euroclone, Pero, MI, Italy). Cultures have been maintained at 37°C in a humidified atmosphere containing 5% CO_2_. After 48-hours adhesion, non-adherent cells were discarded and culture medium was replaced twice a week. MSCs were harvested till the ninth passage (P0–P9), splitted after reaching ≥80% confluence for each passage, using Trypsin (Euroclone, Pero, MI, Italy), and re-plated for expansion at 4,000 cells/cm^2^. MSC mRNA and proteins were obtained for each passage from P0 to P9.

MSCs were then characterized by flow cytometry using FITC or PE-conjugated monoclonal antibodies specific for the following antigens: CD45, CD14, CD34, CD13, CD80, CD31, HLA A-B-C, HLA-DR, CD90 (BD PharMingen, San Diego, CA), CD73, CD105 (Serotec, Kidlington, Oxford, UK) (Figure S1). Appropriate, isotype-matched, non-reactive fluorocrome-conjugated antibodies were employed as controls. Analysis of cell populations was performed by means of direct immunofluorescence with a FACSCanto flow-cytometer (BD PharMingen) and data were elaborated using the FACSDiva software (TreeStar Inc., Ashland, OR).

### Molecular analysis

#### RNA isolation

After cell cultures were trypsinized, mRNA extraction was performed by using a RNA Tri-Reagent (Molecular Research Center Inc., Cincinnati, OH), according to the manufacturer's instructions. RNA concentrations were determined by UV spectrophotometer (NanoDrop ND 1000, NanoDrop Technologies, LLC, Wilmington, DE).

#### Retrotranscription, semiquantitative PCR, Real-Time PCR

Reverse transcriptase from Avian Myeloblastosis Virus (Promega, Madison, WI) was used. Specific amplification reactions for CB1 (alias *CNR1*, GeneID1268), CB2 (alias *CNR2* GeneID1269), and for the housekeeping β-actin cDNA were carried out. Amplimers were resolved into 2.0% agarose gel, detected by the Gel Doc 2000 UV System (Bio-Rad, Hercules, CA, USA).

Three serial 2x cDNA dilutions obtained from 250 ng total mRNA were amplified by Real-Time PCR, using SYBR green as fluorophore, in order to quantify the expression levels of CB1 and CB2 with respect to β-actin. Assays were performed in triplicate. Twenty-five µl reaction contained: 2 µl cDNA, 12.5 µl SYBR green Master Mix (Bio-Rad, Hercules, CA, USA), 10 µl primers mix (10 mM). The thermal cycling program was: 95°C-10 min, followed by 40 cycles of 95°C-15 s and 60°C-1 min. Gene expression profiling was achieved using the comparative cycle threshold method of relative quantization to the housekeeping gene. Real-Time PCR products were analyzed by Icycler software (Bio-Rad, Berkeley, USA).

### Biochemical analysis

#### Western blot

Total lysates from MSC cultures obtained through RIPA buffer lysis were analyzed by western blot (WB) experiment at some of the different passage from P1 to P9 and after pharmacological challenge with the CB2 agonist JWH-133 or the CB2 antagonist AM630. Fifty micrograms of denaturated protein were loaded. Membrane strips were alternatively incubated overnight at 4°C with the following horseradish peroxidase-conjugated antibodies: rabbit polyclonal anti-CB2 (1∶200; Abcam, Cambridge, UK), p-AKT (1∶500), p-ERK1/2 (1∶500), pS6K1 (1∶800), BCL2 (1∶800), IL-10 (1∶500) (Santa Cruz Biothecnology, Santa Cruz, CA), and then with the relative secondary antibody for 1 hour; reactive bands were detected by chemiluminescence (Pierce SuperSignal West Femto, Rockford, IL) on a ChemiDoc station (Bio-Rad, Hercules, CA). Whereas the same membrane strip was used for revealing the expression of more than one protein of interest, a mild stripping at 60°C for 10 minutes was done. Monoclonal anti-β-tubulin antibody (1∶1000 or 1∶2000; Sigma, Milan, Italy) was used as housekeeping protein to check for identical protein loading.

#### Immunocytochemistry

Specific antibodies against CB2 and CD105 have been used to characterize hMSCs at different passages from P0 to P9. Human MSCs, harvested in specific chamber slides, were fixed for 10 minutes with PFA 4% and then washed with PBS 1X. Cells were then incubated for 3 hours with primary antibodies for CB2 (rabbit anti-CB2 abcam 1∶250, Cambridge, UK) and CD105 (mouse anti-CD105, Millipore, EMD Millipore Corporation, MA, USA), or with a mixture of CB2 (goat anti-CB2, Santa Cruz Biothecnology, Santa Cruz, CA, USA, diluted 1∶100) and pS6K1 (rabbit anti-ribosomal S6 kinase, Cell Signaling Technology, Boston, MA, USA; diluted 1∶800) and after 3 washes with PBS were re-incubated 1 hour with secondary antibodies (Alexa fluor 488 and 568). The cells on chamber slides were mounted and visualized as containing DAPI through epifluorescence with a Leica DMI6000 microscope.

#### Endocannabinoid extraction and quantification

MSCs at different passages (P0–P9) and their supernatants were collected in tubes and extracted with an equal volume of methanol/chloroform (1∶2 by vol.) containing 5 pmol of deuterated AEA and 2-AG, as well as deuterated N-oleoylethanolamine (OEA) and N-palmitoylethanolamine (PEA), two AEA congeners with little activity on cannabinoid receptors but endowed with anti-inflammatory activity. Lyophilized lipid extracts were then re-suspended in chloroform/methanol (99∶1, by vol.) and the solutions purified by open bed chromatography on silica, as previously described [29]. Fractions eluted with chloroform/methanol (9∶1, by vol.) and containing AEA, PEA, 2-AG and OEA, were collected and the excess solvent evaporated in a rotating evaporator; aliquots were analysed by isotope dilution-liquid chromatography/atmospheric pressure chemical ionization/mass spectrometry (MS) carried out under conditions described previously [29] and allowing the separations of 2-AG, PEA, AEA and OEA. Results were expressed as pmol/mg of extracted lipids.

#### ELISA

The hMSC culture supernatant levels of IL-1β, IL-6, IL-8, IL-10, IL-12, IL-17, TNF-α and INF-γ were measured using a commercially available Human Inflammatory Cytokines Multi-Analyte ELISArray Kit (Qiagen, QIAGEN S.p.A., Italy). The assay was performed according to the manufacturer's instructions. Briefly, a microplate was coated with a monoclonal antibody that was specific for each of the seven investigated cytokines. Standards (antigene mix for all the investigated cytokines) and hMSC supernatants were pipetted into the wells. Supernatants were obtained from four different hMSC cultures: untreated; treated with 500 ng/ml LPS; treated with the specific CB2 agonist JWH-133 1 µM; co-treated with LPS and JWH-133. Human MSCs were treated at passage 6 (P6), when the expression of CB2 is high. A positive control was obtained pipetting into the wells only the standard mix. A negative control was obtained by pipetting standards and supernatants into non-coated wells. After washing, an enzyme-linked polyclonal antibody specific for each different cytokine was added. The reaction was revealed by addition of the substrate solution. The optical density was measured at a wavelength of 450 nm by using the Tecan Infinite M200 (Tecan, Switzerland) spectrophotometer. The serum concentration of the different cytokines (pg/ml) was determined against a standard concentration curve. The correlation coefficient (r) of the standard concentration curve was 0.990. Values with a coefficient of variation beyond 10% were not included in the final data analysis. All samples were run in duplicate.

### hMSC migration assay

The attractive properties of 2-AG were analyzed in a modified chemiotaxis plate (iuvo Chemotaxis Assay plate, Thermo Scientific Inc., Germany). One hundred microliters of 1–2.5×105 P6 hMSCs per cm2 were seeded in 5 mm cell port 24 hours before the migration assay. Twenty microliters of assay medium containing either CB2 selective inhibitor AM630 [2 µM] or vehicle were added to the attractant port 15 min before the addition of 2-AG [10 µM]. Then 20 µL 2-AG (×106 diluted stock in ethanol) were added to the attractant port permeating through the unit and creating a chemo attractant gradient in the gradient channel. The concentration of 2-AG [10 µM] used for the migration assay derived by previous experiments in which three different concentrations have been used (Figure S2). A source of HGF (PeproTech, UK) was used as positive control.

Using DAPI image-based detection the number of migrating cells was determined counting the migrated cells from the plates where hMSCs were exposed to vehicle or 2-AG chemo attractant ligand or AM630 before 2-AG treatment.

### Drugs and treatments

LPS, AM630, JWH-133 and AM251 (Tocris, Avonmouth, UK) were dissolved in PBS containing DMSO. DMSO final concentration on cultures was 0.01%. Cultured hMSCs were treated with AM630 [2 µM], JWH-133 [100 nM or 1 µM], or 500 ng/ml LPS. JWH-133 and LPS, alone or in combination were applied for 24 hours, AM630 was applied for 15 min before JWH-133 treatment. Non-treated cultured MSCs were maintained in incubation media during the relative treatment time with or without vehicle (DMSO 0.01%). Any significant difference was observed between not treated and vehicle-treated cells. Figures show vehicle data.

### Statistics

Molecular and biochemical data are shown as means ± SD. All the experiments were at least in triplicate. A t-test was performed according to the number of experiments. A *p* value less than 0.05 was considered statistically significant.

## Results

### Expression of CB1 and CB2 receptors in human MSCs

**Figure 1:**
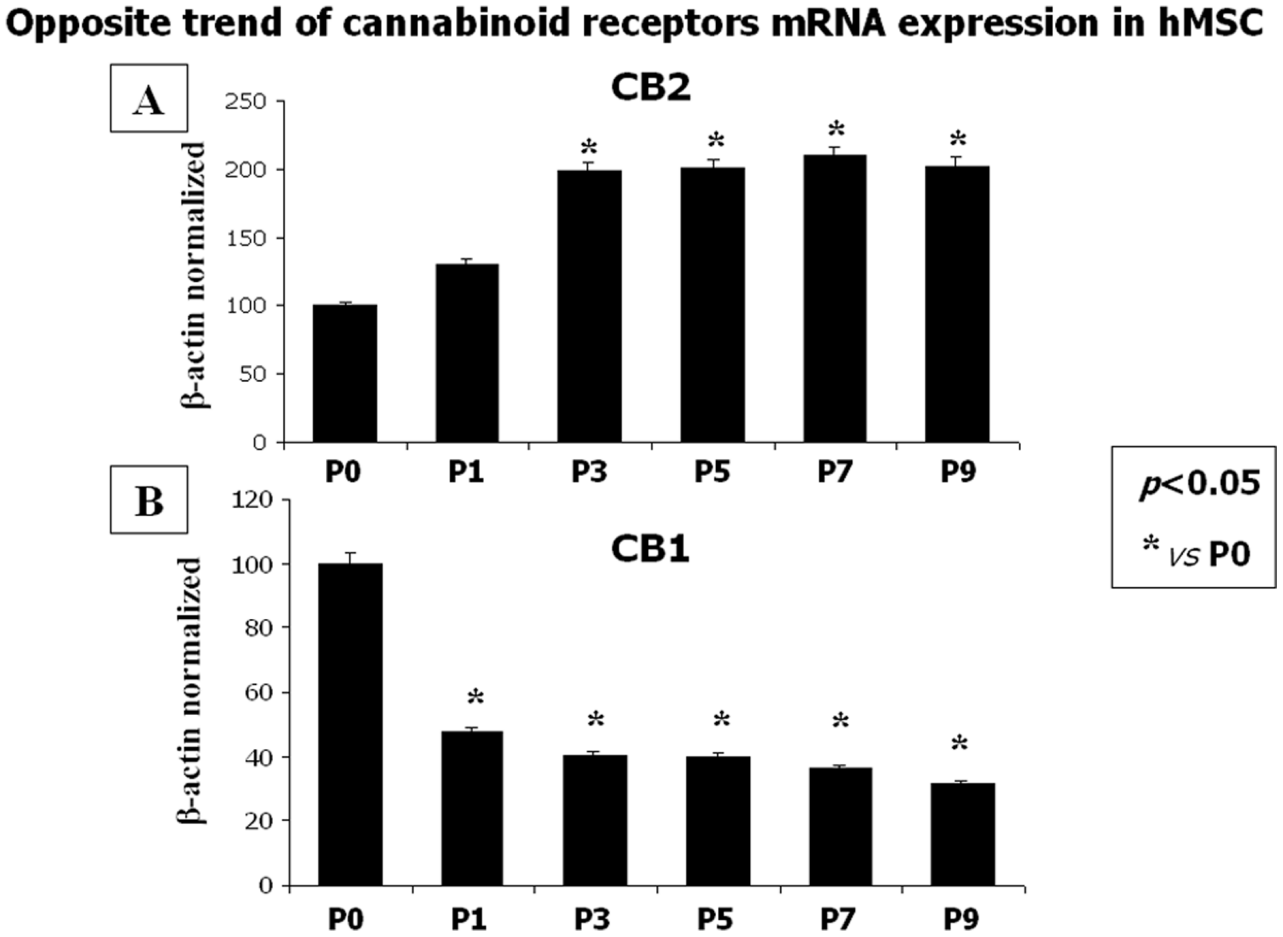
Expression of CB1 and CB2 receptors in human MSCs. (**A**) Significant increase of Cannabinoid Receptor type 2 (CB2) expression levels in human Mesenchymal Stromal Cells from passage 3, P3. (**B**) Cannabinoid Receptor type 1 (CB1) expression levels showed a significant decrease starting from passage 1. Data are revealed from human in vitro MSCs by real time PCR by using three serial dilution 2X starting from 250 ng of total mRNA for the RT reaction and normalized for the housekeeping gene β-actin. Assays are at least in triplicate. Data are represented as a mean ± SD. A t-test has been used for statistical analysis. *p*<0.05 was considered statistically significant.

RT-PCR followed by cDNA amplification demonstrated the presence of mature mRNA for CB1 and CB2 receptors in hMSCs. We found that the expression of the CB2 receptor was significantly lower at the first 2 passages as compared to the following ones. In particular, the highest amount of CB2 mRNA was observed starting from P3 (Fig. 1A). An opposite trend was observed for the CB1 receptor expression, which significantly decreased since P1 (Fig. 1B).

### CB2 expression level in hMSCs increases with their maturation

**Figure 2:**
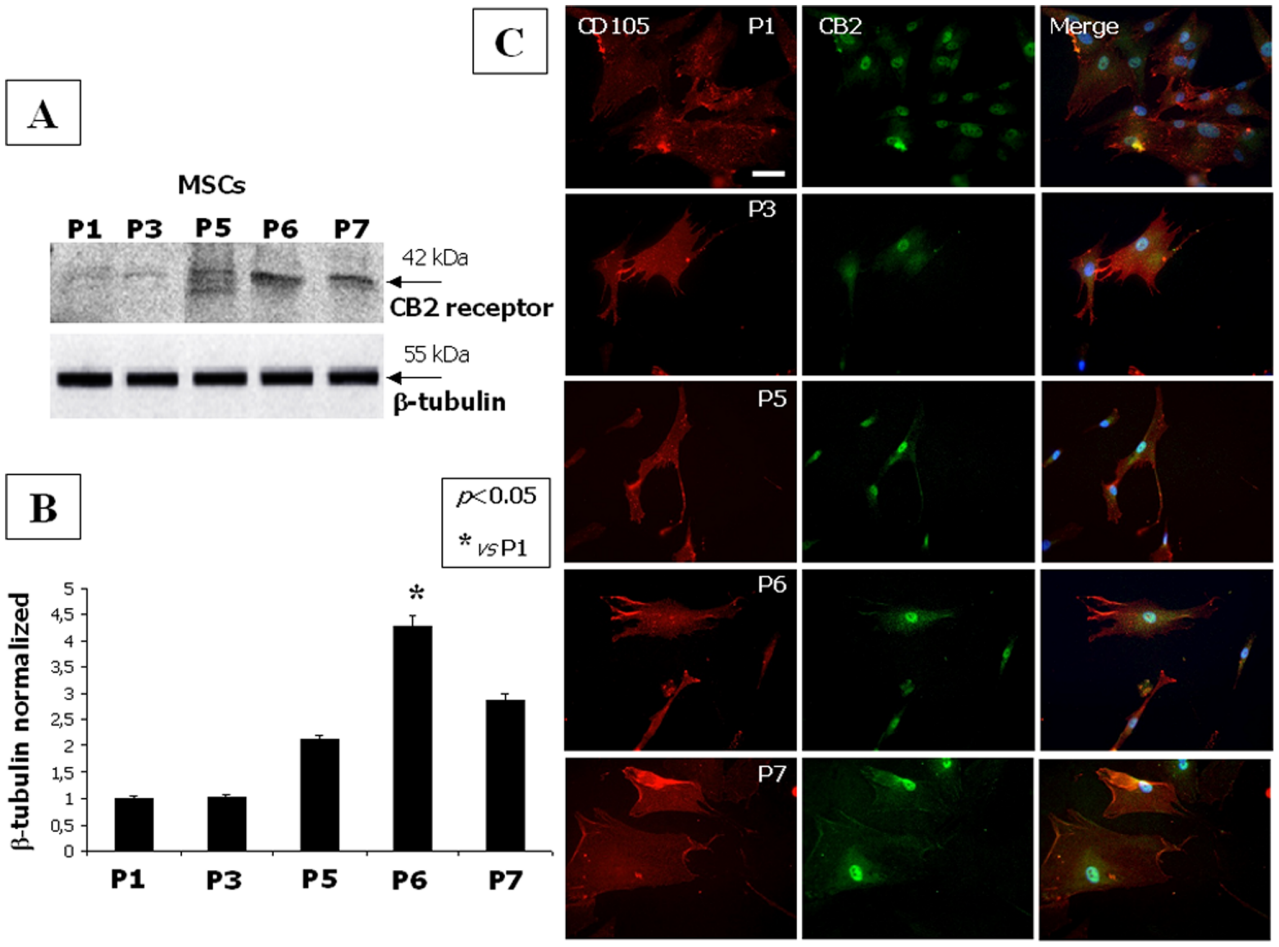
CB2 expression level in hMSCs increases with their maturation. (**A**) Western Blot experiment and (**B**) the relative quantification for CB2 receptor expression in total MSC lysates from different passages normalized with respect to β-tubulin reveal a significant increase of the protein levels at P5 with a maximal expression at P6. Data are represented as a mean ± SD from three different assays. A t-test has been used for statistical analysis. *p*<0.05 was considered statistically significant. (**C**) A positive staining for the specific mesenchymal CD105 marker (in red) and an increased CB2 receptor expression from passage P1 to P7 (in green) is highlighted by immunocytochemistry analysis. Scale bar 50 = 50 microns.

The CB2 protein level was investigated by western blot and immunocytochemistry (Fig. 2).

The protein level of CB2 receptor was found to be up-regulated in lysates of hMSCs from passage P5, reaching the maximum expression at P6 (Fig. 2A–B).

CB2 staining on MSCs, labeled with CD105 mesenchymal marker, was up-regulated at P6 (Fig. 2C).

### Endocannabinoid levels

**Figure 3:**
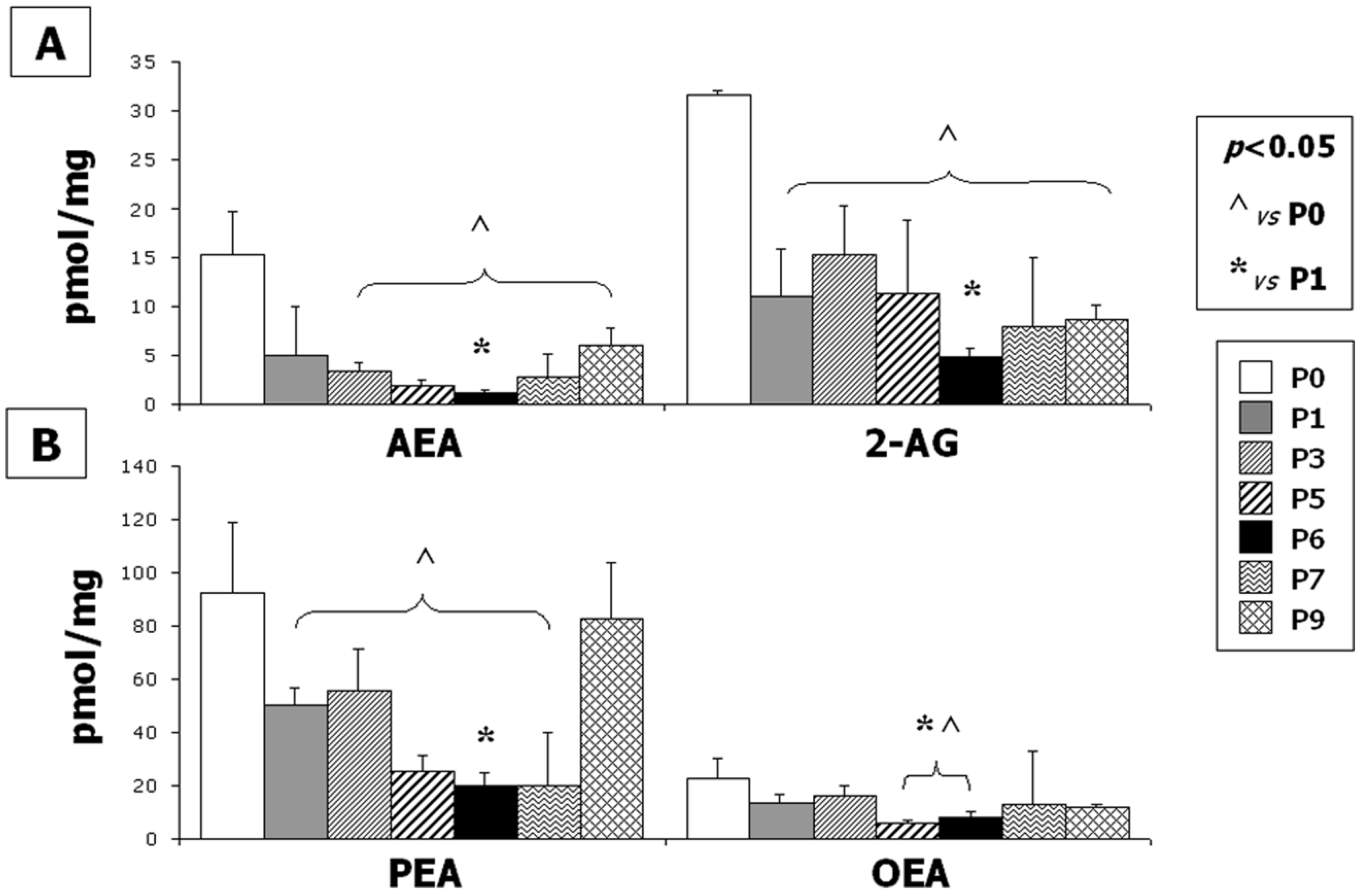
Endocannabinoid levels. Human mesenchymal stem cells (MSCs) contained measurable amounts of AEA, 2-AG (A), PEA and OEA (B). A significantly decrease of all the endocannabinoids was detected during expansion passages from P1 to P9 with respect to P0. The minimum amount of AEA, 2-AG and PEA was detected at P6 (AEA 1.26±0.27 pmol/mg extract; 2-AG 4.79±0.81 pmol/mg extract; PEA 20.10±4.76 pmol/mg extract). The minimum amount of OEA was detected at P5 (AEA 6.13±1.11 pmol/mg extract). Data are represented as a mean ± SD from N = 6 experiments. A t-test has been used for statistical analysis. *p*<0.05 has been considered statistically significant.

Human MSCs at different passages contained measurable amounts of AEA, 2-AG (Fig. 3A), PEA and OEA (Fig. 3B). AEA levels were higher at P0-1 (P0 and P1) and decreased throughout the subsequent expansion passages. The minimum amount of AEA, 2-AG and PEA was detected at P6 (AEA 1.26±0.27 pmol/mg extract; 2-AG 4.79±0.81 pmol/mg extract; PEA 20.10±4.76 pmol/mg extract). The minimum amount of OEA was detected at P5 (AEA 6.13±1.11 pmol/mg extract) (Fig. 3 A, B).

### Stimulation of the CB2 receptor partially reverses the LPS-induced modulation of pro- and anti- inflammatory cytokines in hMSCs

**Figure 4:**
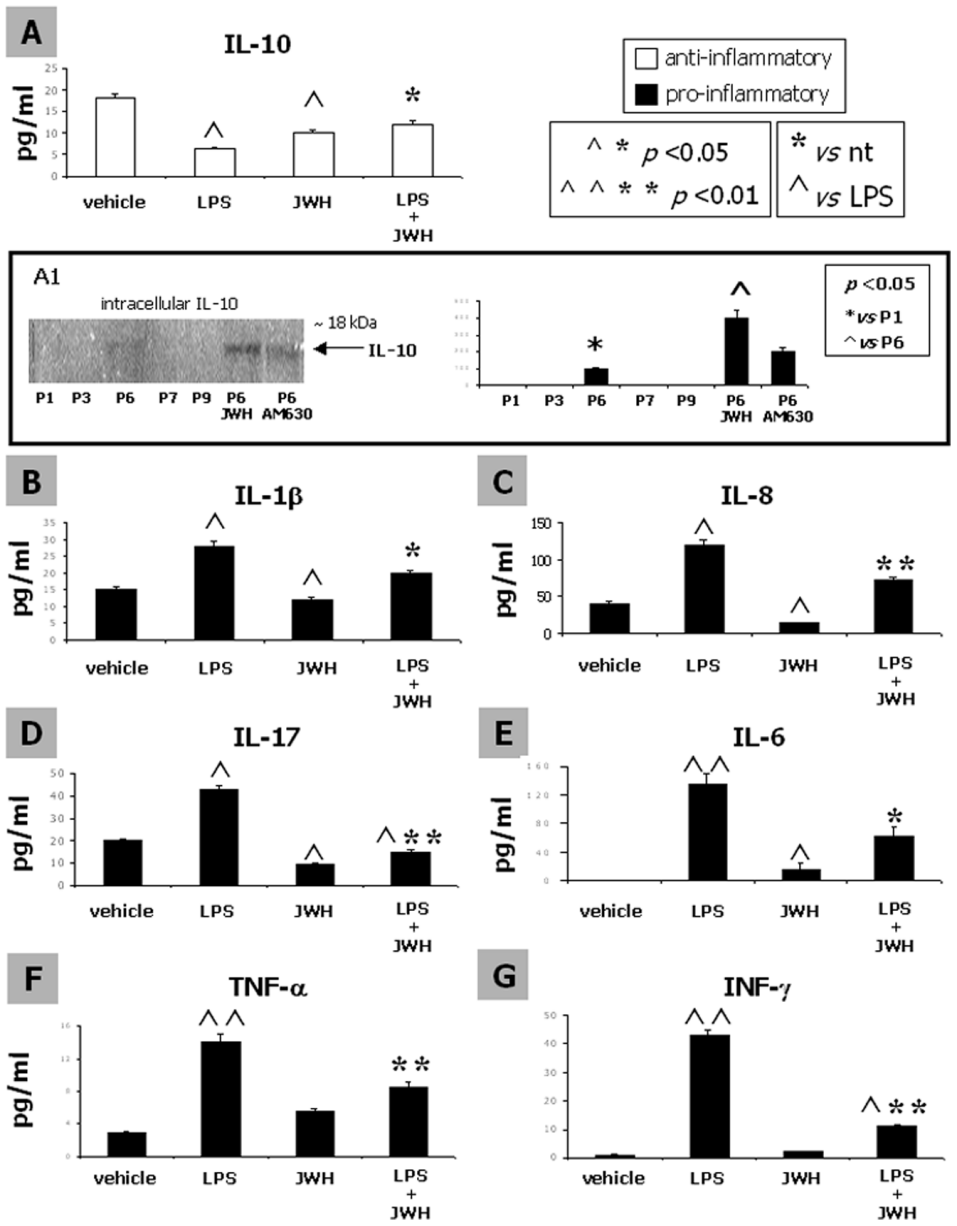
Stimulation of the CB2 receptor partially reverses the LPS-induced modulation of pro- and anti- inflammatory cytokines in hMSCs. The release of the anti-inflammatory IL-10 (A) and of the pro-inflammatory IL-1β (B), IL-8 (C) and IL-17 (D), as well as of IL-6 (E), TNF-α (F) and INF-γ (G) from MSCs have been investigated through a multi-ELISA assay. IL-10 secretion by MSC cells was significantly reduced in response to 500 ng/ml LPS, while IL-1β, IL-8 and IL-17 were significantly increased. The treatment with the CB2 agonist JWH.-133 1 µM was able to revert the LPS-induced effect. The CB2 stimulation decreased the release of all the cytokines, even of the anti-inflammatory IL-10, thus was conversely shown to be up-regulated in total cellular lysates both at P6 and after CB2 stimulation, as revealed by the western blot included in the A1 inset. The release of IL-6, TNF-α and INF-γ by MSC cells, undetectable at basal condition, was significantly increased in response to 500 ng/ml LPS. The treatment with the CB2 agonist JWH-133 1 µM was able to fully revert the LPS-induced effect. Mean concentration ± SD (pg/ml) for all the cytokines from triplicate cultures is shown. A t-test has been used for statistical analysis. *p*<0.05 has been considered statistically significant.

The ELISA assay revealed that LPS induced a reduction in the release of the anti-inflammatory cytokine IL-10 (Fig. 4A), and, conversely, a significant enhancement of the pro-inflammatory cytokines IL-1β (Fig. 4B), IL-8 (Fig. 4C) and IL-17 (Fig. 4D).

CB2 activation with JWH-133 reversed the LPS-induced effect on both pro- and anti-inflammatory analysed cytokines (Fig.4A–D).

The ELISA showed also a JWH-133-induced reduction of IL-10 release, while a western blot for IL-10 on total lysate demonstrated that JWH-133 increased IL-10 expression (Fig. 4, A1 inset)

The pro-inflammatory cytokine IL-6 was significantly secreted in supernatant of hMSC cultures treated with LPS as compared to vehicle-treated cells, which did not release detectable amounts of IL-6 levels (Fig. 4E). Similarly, TNF-α (Fig. 4F) and INF-γ (Fig. 4G) were detectable in minimal amounts in vehicle-treated cells, whereas their level significantly increased after the LPS challenge. The secretion of the pro-inflammatory cytokines IL-6, TNF-α and INF-γ was fully reverted by the selective CB2 agonist JWH-133.

### Stimulation of CB2 directly stimulates cell survival pathways

**Figure 5:**
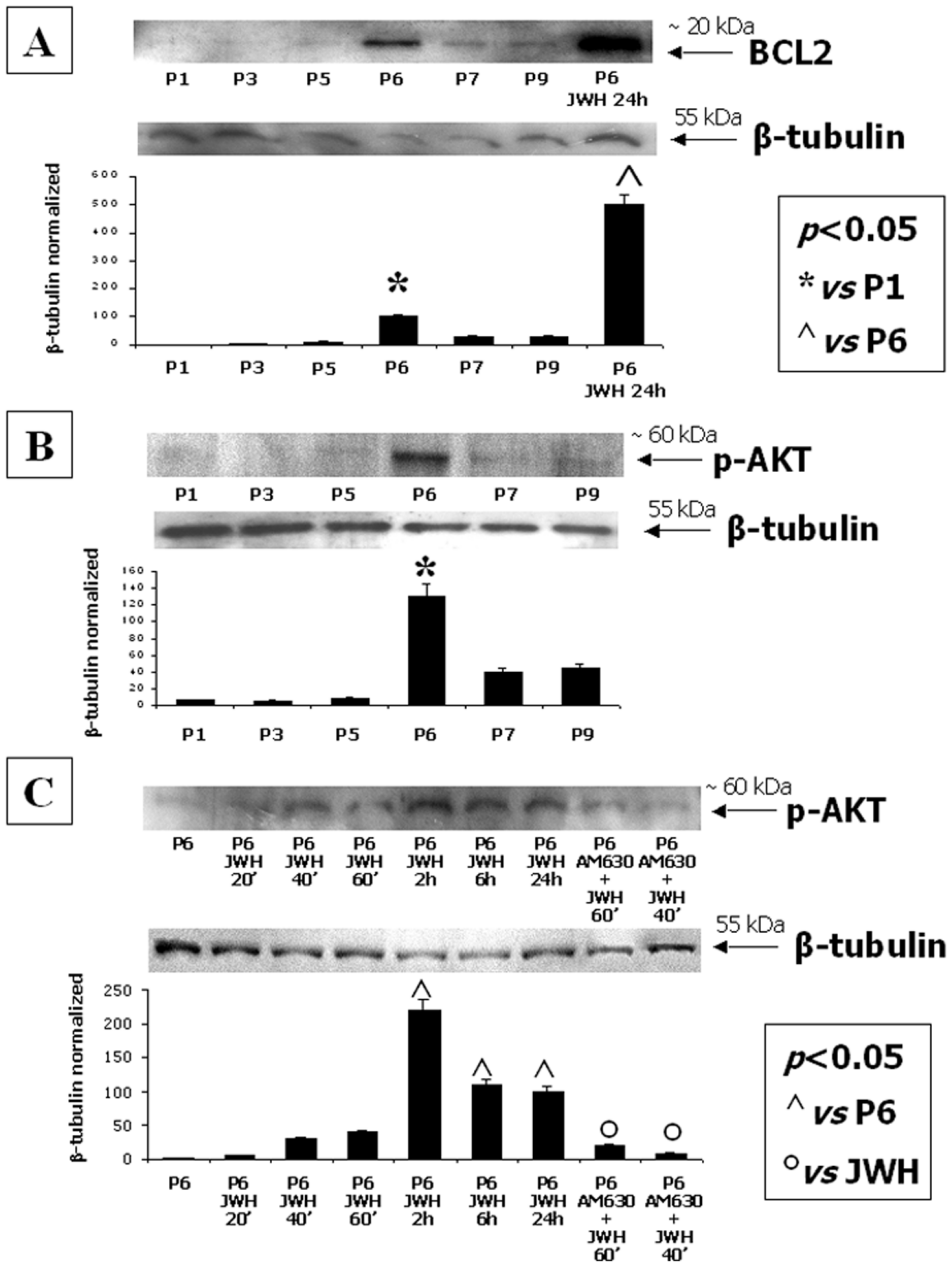
Stimulation of CB2 directly stimulates cell survival pathways. (**A**) Western Blot experiment and the relative quantification for the anti-apoptotic factor BCL-2 expression in total MSC lysates from different passages normalized with respect to β-tubulin reveal a significant increase of the protein levels at P6 and after CB2 selective stimulation. (**B**) Western Blot experiment and the relative quantification for p-AKT expression normalized with respect to β-tubulin in total MSC lysates from different passages and (**C**) after CB2 selective stimulation at multiple time-points. Data show the maximum increase of the protein levels at P6 and after 2 hours from CB2 selective stimulation with the synthetic agonist JWH-133. The CB2 antagonist AM630 reduces the JWH-induced increase of p-AKT. Data are represented as a mean ± SD from three different assays. A t-test has been used for statistical analysis. *p*<0.05 was considered statistically significant.

Western blot of hMSC total lysates revealed that the anti-apoptotic factor BCL2 was significantly higher at P6, with respect to other passages, and also after incubation with the CB2 agonist JWH-133 (Fig. 5A). Accordingly, the survival pathway was more activated at P6 and after CB2 selective stimulation, as revealed by pAKT protein level changes (Fig. 5B, C). The CB2 antagonist AM630 reduced the up-regulation of the p-AKT induced by JWH-133.

### Stimulation of CB2 directly stimulates the ERK2 pathway

**Figure 6:**
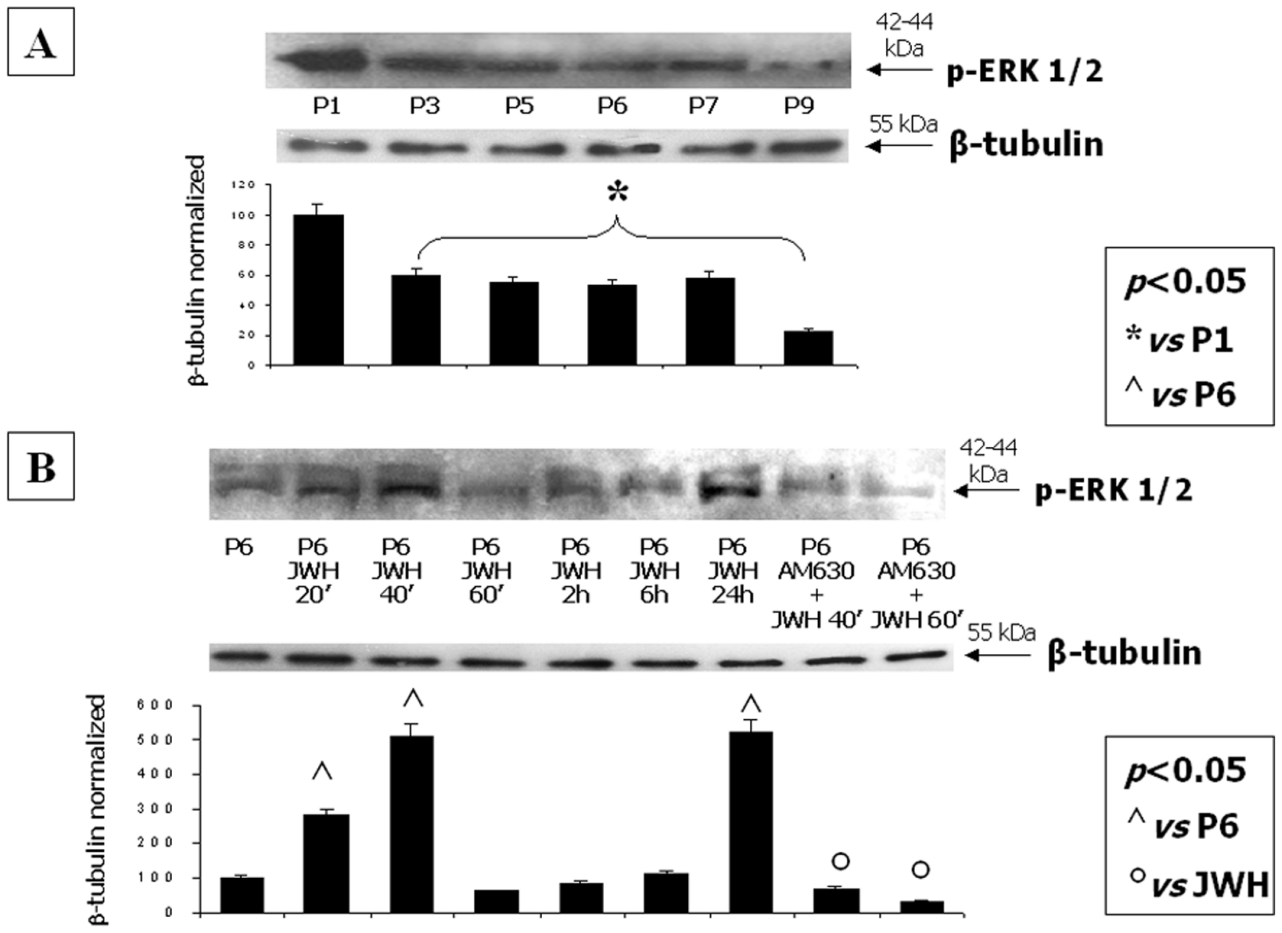
Stimulation of CB2 directly stimulates the ERK2 pathway. (**A**) Western Blot experiment and the relative quantification for p-ERK2 expression normalized with respect to β-tubulin in total MSC lysates from different passages and (**B**) after CB2 selective stimulation at multiple time-points. Data show the maximum increase of the protein levels at P1 and after 20-40 minutes and 24 hours from CB2 selective stimulation with the synthetic agonist JWH-133. The minimal p-ERK2 expression is revealed at P6. The CB2 antagonist AM630 significantly counteracts the JWH-induced increase of p-ERK2. Data are represented as a mean ± SD from three different assays. A t-test has been used for statistical analysis. *p*<0.05 was considered statistically significant.

Western blot of hMSC total lysates at different passages from P0 to P9 (Fig. 6A) and at different times after CB2 stimulation revealed that phosphorylated ERK2 was preferentially expressed with respect to p-ERK1. In particular, p-ERK2 level was significant higher at P1, with respect to other passages, (Fig. 6A) and both at 40 minutes and 24 hours after incubation of the cells with the CB2 agonist JWH-133 (Fig. 6B). The CB2 antagonist AM630 significantly counteracted the increase of p-ERK2 induced by JWH-133.

### CB2 selective stimulation is associated with mTOR pathway activation

**Figure 7:**
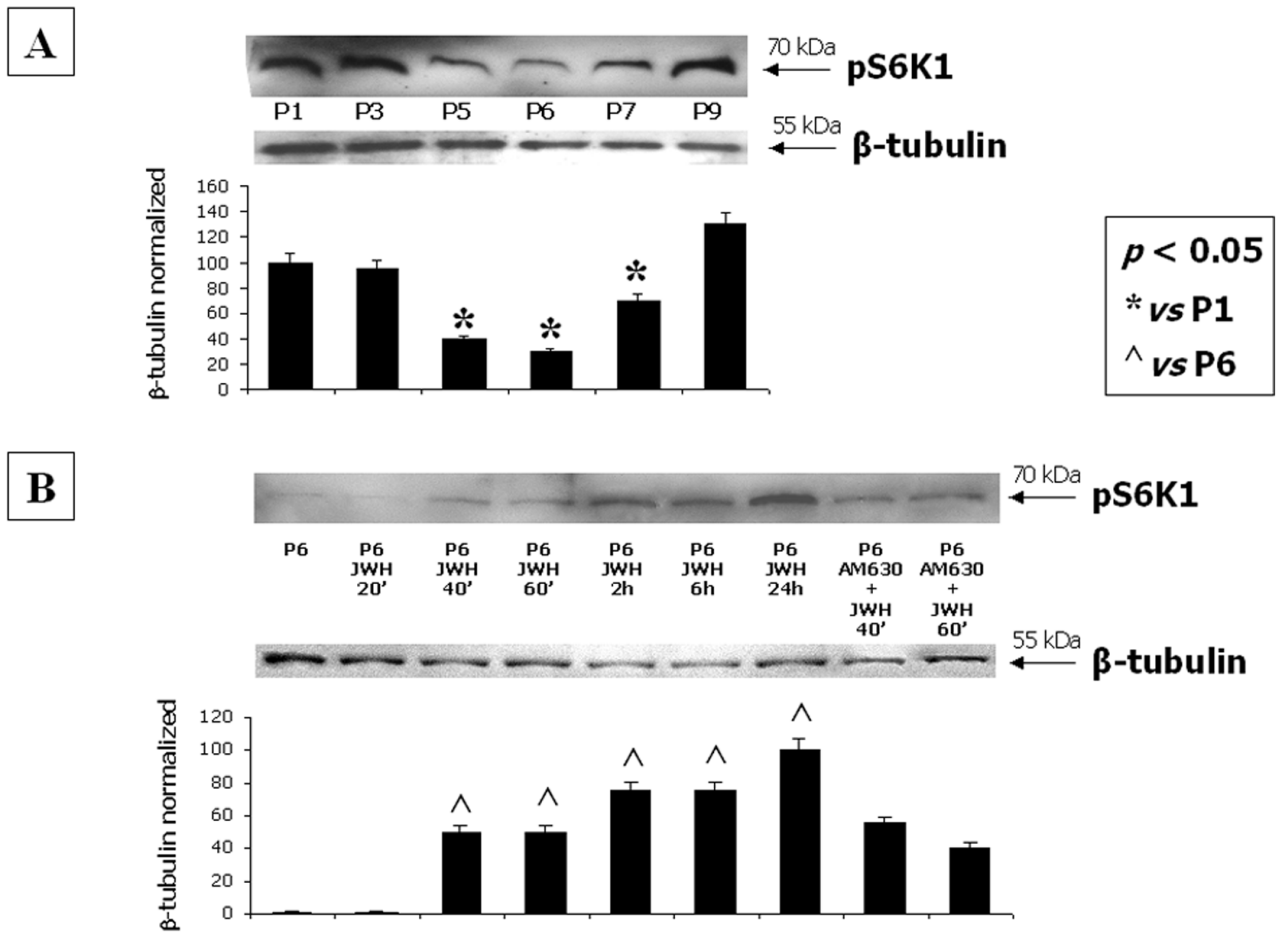
CB2 selective stimulation is associated with mTOR pathway activation. (**A**) Western Blot experiment and the relative quantification for pS6K1 expression normalized with respect to β-tubulin in total MSC lysates from different passages and (**B**) after CB2 selective stimulation at multiple time-points. Data show the maximum increase of the protein levels at P1 and P9 and after 24 hours from CB2 selective stimulation with the synthetic agonist JWH-133. The minimal expression is revealed at P6. The CB2 antagonist AM630 reduces the JWH-induced increase of p-AKT. Data are represented as a mean ± SD from three different assays. A t-test has been used for statistical analysis. *p*<0.05 was considered statistically significant.

Activation of the mTOR pathway, as revealed by phospho-S6K1 levels, pS6K1, was higher at first and last harvesting passages, as compared to P6. Nevertheless, the CB2 selective stimulation of MSCs at P6 significantly increased pS6K1 levels during the two hours from drug treatment, reaching the maximum after 24 hours (Fig. 7). The CB2 antagonist AM630 reduced the up-regulation of the p-S6K1 induced by JWH-133.

### 2-AG is Chemoattractant for hMSCs through CB2 receptor

**Figure 8:**
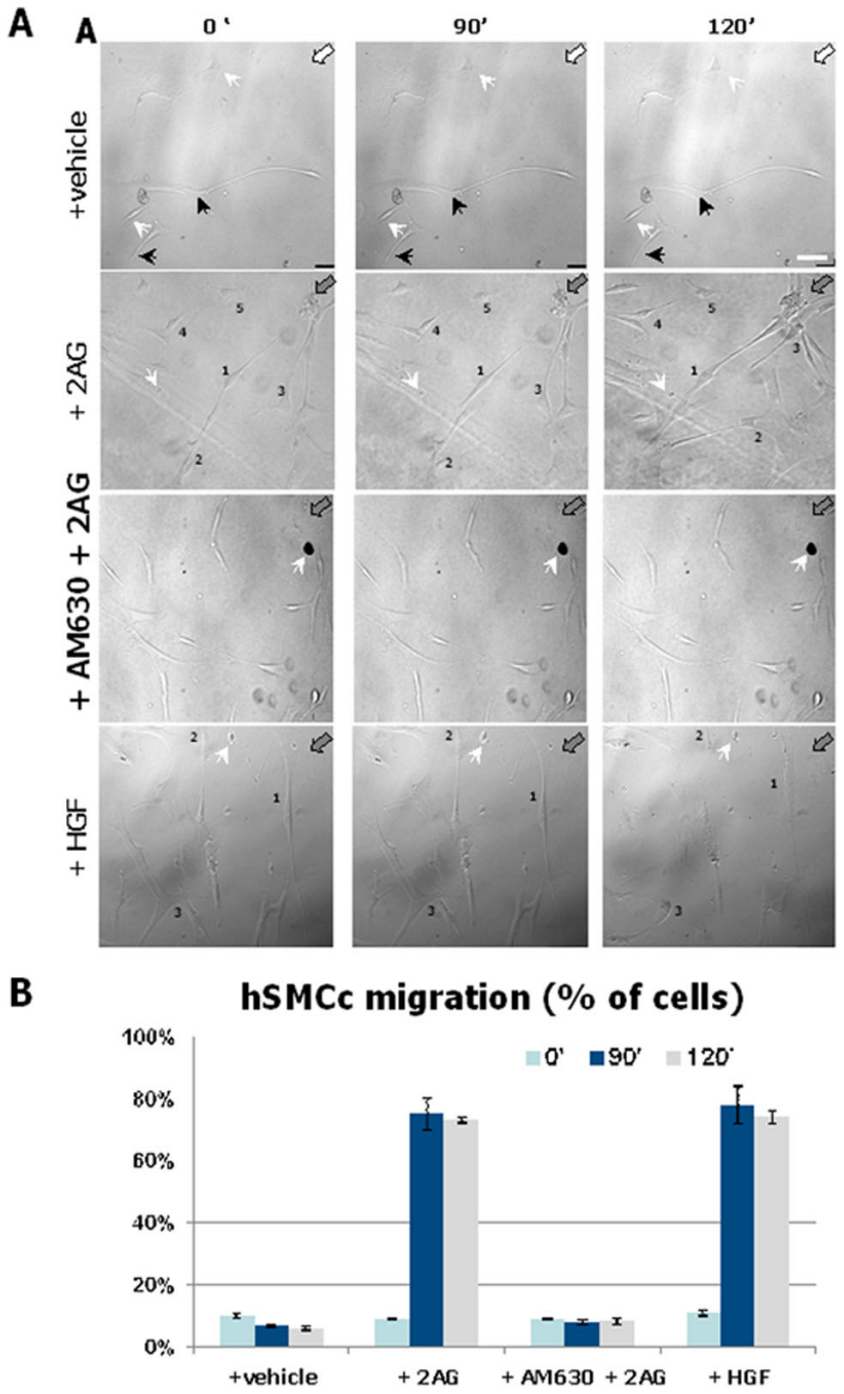
2-AG is Chemoattractant for hMSCs through CB2 receptor. (A) Image-based detection of 2-AG chemoattractive effect on cultured hMSCs. Representative morphological data of hMSCs time-lapse migration recorded at starting point (0 min), 90 min and 120 min from the 2-AG (filled gray arrows) or vehicle (white arrows) exposure. The cells were maintained in the relative medium or pretreated with the CB2 antagonist AM630 (lower panel) 15 min before the exposure to 2-AG during the chemotaxic assay. The arrowheads indicate static reference points in the cell port chamber; the black arrows point to soma or cellular processes without noticeable movements. The numbers indicate the hMSCs or their processes affected by 2-AG chemoattractive movements. Scale bar = 30 microns. A source of HGF has been used as positive control. (B) Quantative data of percentage of hMSCs effected by chemotaxic movements. n = 300 cells per each treatment. *** *P*<0.0001 of hMSCs migration toward 2-AG at 90 min and 120 min *versus* hMSCs migration at each different treatment and time course.

We observed maximal migration of hMSCs toward the [10 µM] 2-AG concentration, in the same extent of HGF-induced migratory effect used as a positive control. Treatment with the specific CB2 antagonist AM630 blocked 2-AG-induced chemotaxis, confirming the involvement of CB2 activation in this process (Fig. 8).

### mTOR/S6K1 pathway is involved in the hMSCs migration 2AG-mediated

**Figure 9:**
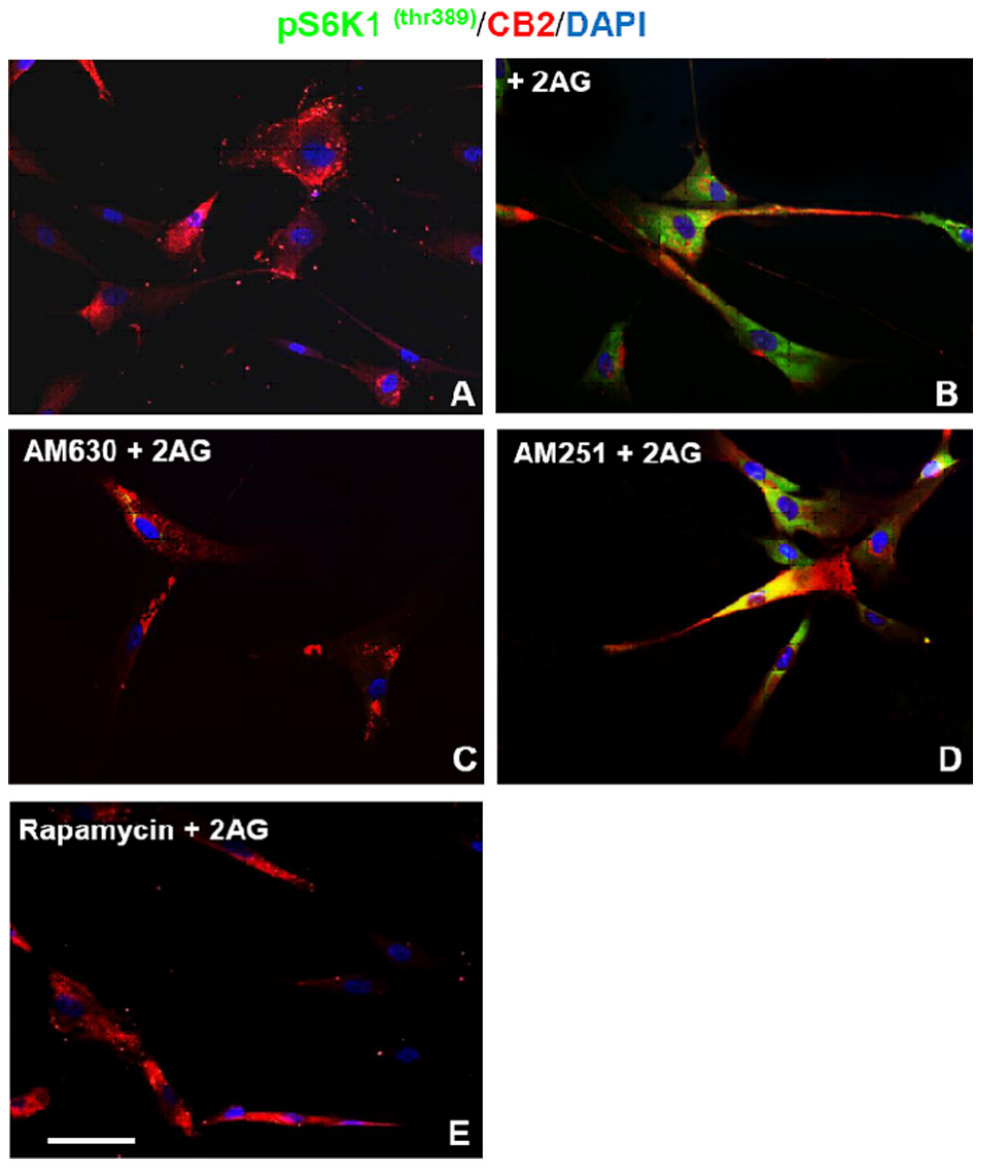
mTOR/S6K pathway is involved in the hMSCs migration 2AG-mediated. Immunocytochemical expression of the phosphorylated S6K1thr389 in the cytoplasm of hMSCs after 1h of exposure in absence (**A**) or presence (**B**) of 2-AG [10 µM] in the medium. The pS6K1 thr389 expression is strongly reduced by pretreatment of hMSCs with CB2 antagonist AM630 [2 µM] 15 min before the 2AG addition (**C**) whereas is unaffected by similar pretreatment with CB1 antagonist AM251(**D**). The Mammalian Target of Rapamycin (mTOR)-dependent action on 2-AG-induced phosporylation of pS6K1 thr389 in hMSCs is prevented by addition of rapamycin in medium 15 min before the 2AG addition (**E**). Scale bar = 10 microns.

Immunocytochemical expression of the phosphorylated S6K1thr389 in the cytoplasm of hMSCs was strongly induced after 1 h of exposure to 2-AG [10 µM] in the medium (Fig. 9B). On the contrary, the phosporylation of pS6K1 thr389 2-AG-induced, was strongly inhibited after CB2 antagonist AM630 [2 µM] cellular treatment 15 min before the 2-AG addition in the medium (Fig. 9C) or after inhibition of the mTOR/S6K pathway through rapamycin (Fig. 9E) whereas was unaffected by CB1 antagonist AM251(Fig. 9D). These results demonstrate the specific activity of CB2 on the Mammalian Target of Rapamycin (mTOR)-dependent action of 2-AG-induced phosporylation of pS6K1 thr389 in hMSCs.

## Discussion

Homeostasis inside BM is sustained by a complex interaction of growth factors, cytokines and transcription factors that orchestrate the proliferation and differentiation of stem cells (i.e. hematopoietic stem cells [HSCs]; MSCs; endothelial progenitor/stem cells [EPCs]) into one or more cell types. It has been shown that BM stromal cells synthesize and release *on demand* endocannabinoids and express their specific G protein-coupled receptors CB1 and CB2. Specifically, there is evidence that stimulation of CB2 receptor plays a critical role in hematopoiesis and cell mobilization, this finding pointing to the endocannabinoids as novel mediators for governing tissue migration and homing of HSCs *in vivo* [26].

In this study, we demonstrate, to the best of our knowledge for the first time, the presence and regulation of the endocannabinoid system in hMSCs and its possible involvement in their anti-inflammatory/immunomodulatory activity. We found that hMSCs express both CB1 and CB2 receptors (Fig. 1, 2), as well as the biochemical enzymatic machinery for the synthesis and degradation of the endocannabinoids anandamide (AEA) and 2-arachidonoylglycerol (2-AG) (not shown). A significant change in endocannabinoid levels, concurrently with a sensible modification of CB2 or CB1 receptor expression, was observed during the different *in vitro* culture passages (Fig. 3). We show that the expression of the CB2 receptor was barely detectable at the first passage *in vitro* and increased through the following ones, while CB1 expression showed the opposite trend. Interestingly, this inversely correlated regulation, at least in part, mimicked the same trend of receptor expression we have observed in human osteoclasts [29], [30].This opposite trend of the CB1 and 2 receptor expression is in agreement with a growing body of evidence suggesting that the potential neuronal lineage differentiation may be driven by CB1 stimulation, whereas the potential peripheral lineage differentiation seems preferentially mediated by CB2 signalling [25]. However, this opposite receptorial expression seems to be independent from the endocannabinoid levels of AEA and 2-AG, as well as of the other endocannabinoid-like molecules (PEA and OEA) levels, that we found all significantly decreased.

Therefore it is tempting to hypothesize that the endocannabinoid system in hMSCs is regulated in a way to respond: 1) in a paracrine way, i.e. to “external” endocannabinoids, via CB2 receptor activation, to perform specific tasks such as homing, immunosuppression and anti-inflammatory action; and 2) in an autocrine and CB1-mediated way to “internal” endocannabinoids, to carry out yet to be clarified functions.

In particular, we found that selective CB2 stimulation: i) enhanced hMSCs viability; ii) exerted anti-inflammatory activity in LPS-challenged cells; and iii) stimulated cell migration towards a source of 2-AG, altogether these findings supporting the above hypothesis that extracellular endocannabinoids are important paracrine mediators of cell migration and proliferation, as already suggested for microglia, macrophages and sperm cells [13], [31], [32]. In contrast with our findings, Scutt and Williamson [33] have shown that CB1 or CB2 receptors, as analyzed by flow cytometry, were not expressed in rat MSCs grown under non-differentiating conditions. Apart from the differences between species used in that study (human *vs* rat), this discrepancy could be due also to the different methods used (Real-Time PCR, WB *vs* flow-cytometry). In addition, as revealed in this study, the detection of these receptors is largely dependent on the passage at which MSCs are analyzed.

Based on the evidence that BM-derived MSCs express several Toll like receptors (TLRs), in particular the LPS specific target TLR4 [34], we here further investigated the possible function of the CB2 receptor in mediating MSCs anti-inflammatory and/or immunoregulatory effects. We stimulated MSCs with LPS in presence or absence of the CB2 agonist JWH-133 at P6, when the highest amounts of the CB2 protein were found. We found that CB2 activation counteracted the LPS-induced pro-inflammatory and immunostimulatory effects. In particular, we focused our attention on the extracellular levels of IL-10, IL-1β, IL-8, IL-17, IL-6, TNF-α, INF-γ. Moreover, we also investigated the intracellular levels of IL-10, which has been shown to be increased by CB2 activation through the ERK 1/2 pathway [35]. Indeed, while the effects of JWH-133 on pro-inflammatory cytokines replicated results obtained in previous studies with other cell types [13], [36], i.e. the compound inhibited their LPS-stimulated levels and, in some cases also their basal levels, we obtained some unexpected results when examining extracellular IL-10 levels. In particular, we found that, together with the expected reduction of IL-10 induced by LPS, also JWH-133 exerted a similar effect when given to cells in the absence of LPS. This could be due to accidental lack of overlap between the timing of the treatment and that of the detection of protein release by ELISA. Indeed, when we used western blotting analysis of the cellular lysate, we did find a detectable expression of IL-10 only at P6 and the expected significant enhancement following treatment with JWH-133, which was counteracted by a CB2 antagonist. Accordingly, we found that the phosphorylated isoform of ERK2, known to be involved in CB2 stimulation of IL-10 release [33], increased in the JWH-133-treated group, suggesting that also in this cellular system there is ERK-mediated CB2 regulation of this anti-inflammatory cytokine.

Beside the anti-inflammatory property of CB2 stimulation, we also investigated a possible involvement of this receptor in MSC survival, resistance to apoptosis and metabolic activity by analysing the PI3K-AKT-mTOR pathway and the expression of the anti-apoptotic factor BCL-2. Intriguingly, we observed that the highest CB2 expression, as well as its stimulation, corresponded to an enhancement of the anti-apoptotic BCL-2 and the activation of the AKT-mediated survival pathway. In addition, the phosphorylation of the downstream S6K1 effector, which is also correlated to PI3K-AKT-mTOR pathway activation, increased with CB2 receptor stimulation.

In all the investigated pathways, we found that the CB2 antagonist AM630 counteracted the increase in protein levels induced by CB2 stimulation with JWH-133, with higher and significant effect exerted on p-ERK2 and p-AKT as compared to those exerted on p-S6K1. By contrast, in the migration assay, in which the mTOR pathway activation is induced by 2AG exposure, the expression of the p-S6K1 is strongly inhibited by the AM630. This discrepancy could be due to several reasons, including the different nature of the techniques used to investigate p-S6K1 expression (semi-quantitative WB *vs* qualitative IHC), the different pharmacology used for CB2 stimulation (JWH-133 *vs* 2-AG) and the different ability of the cells (at least in term of timing) for metabolizing the two different drugs (synthetic *vs* endogenous cannabinoid). In addition, the CB2 downstream signal is not directly correlated to p-S6K1, but is directly linked to p-AKT, which in fact we found significantly inhibited by the CB2 antagonist.

Taken together these data suggest that at P6 the expression of the CB2 receptor is highest and the cells are possibly more resistant to apoptotic signals, more metabolically activated and, finally more responsive to external stimuli.

To confirm that endocannabinoids could also represent mediators of MSC homing through the CB2 receptor, we performed live imaging studies by releasing 2-AG from a fixed source, and we observed a stimulation of the migration towards the 2-AG source, which was completely blocked by the CB2 receptor selective antagonist AM630. Finally, we showed that S6K1 is a signaling kinase activated during the chemotaxis elicited by 2-AG. Therefore, we demonstrate that 2-AG is a powerful chemoattractant for P6 hMSCs and that the mechanism by which this is accomplished involves activation of S6K1, which in turn could elicit actin polymerization, cell spreading, pseudopodia formation, and chemotaxis by phosphorylation of its target S6 protein.

In conclusion, our data show, for the first time, that cultures of hMSCs express all of the components of the endocannabinoid system and suggest a potential role for the cannabinoid CB2 receptor as a mediator of MSC anti-inflammatory properties, as well as for their survival pathways and their capability to home and migrate towards endocannabinoid sources.

## Supporting Information

Figure S1 **Immunophenotype of culture-expanded MSCs does not change among different culture passages.** Immunophenotype of culture-expanded MSCs from a representative sample, evaluated at different culture passages, from P1 to P9. At each passage, MSCs proved to be positive for CD13 (**A**), CD90 (**B**) and CD105 (**C**) whereas they were negative for CD31 (**A**), CD34 (**B**) and CD45 (**C**). In **D**, it is shown the mean fluorescent intensity calculated at each passage for each type of surface marker. Any significant difference was observed. Assay was performed in triplicate. A t-test has been used for statistical analysis. *p*<0.05 was considered statistically significant.
(TIF)

Figure S2 **2-AG chemoattractant properties.** Panel shows the migratory effect induced by three different concentrations of 2-AG, [2.5 µM], [5 µM] and [10 µM], respectively, recorded at starting point (0 min), 90 min and 120 min from the 2-AG (filled gray arrows) or vehicle (white arrows) exposure.
(TIF)
