# Supplementary figures and images for: The Cannabinoid Receptor Type 2 as Mediator of Mesenchymal Stromal Cell Immunosuppressive Properties
Source: PLoS One. 2013 Nov 27;8(11):e80022. doi: 10.1371/journal.pone.0080022 (PMC3842278; doi:10.1371/journal.pone.0080022)

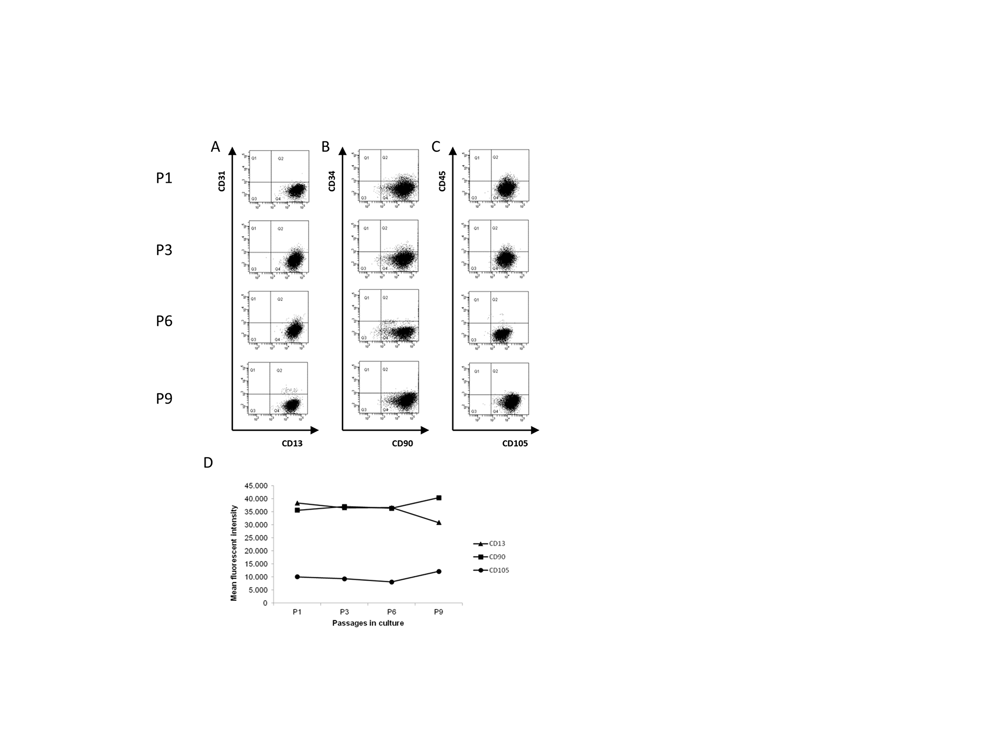

Supplement: Figure S1 — Immunophenotype of culture-expanded MSCs does not change among different culture passages. Immunophenotype of culture-expanded MSCs from a representative sample, evaluated at different culture passages, from P1 to P9. At each passage, MSCs proved to be positive for CD13 (A), CD90 (B) and CD105 (C) whereas they were negative for CD31 (A), CD34 (B) and CD45 (C). In D, it is shown the mean fluorescent intensity calculated at each passage for each type of surface marker. Any significant difference was observed. Assay was performed in triplicate. A t-test has been used for statistical analysis. p<0.05 was considered statistically significant. (TIF) [file pone.0080022.s001.tif]

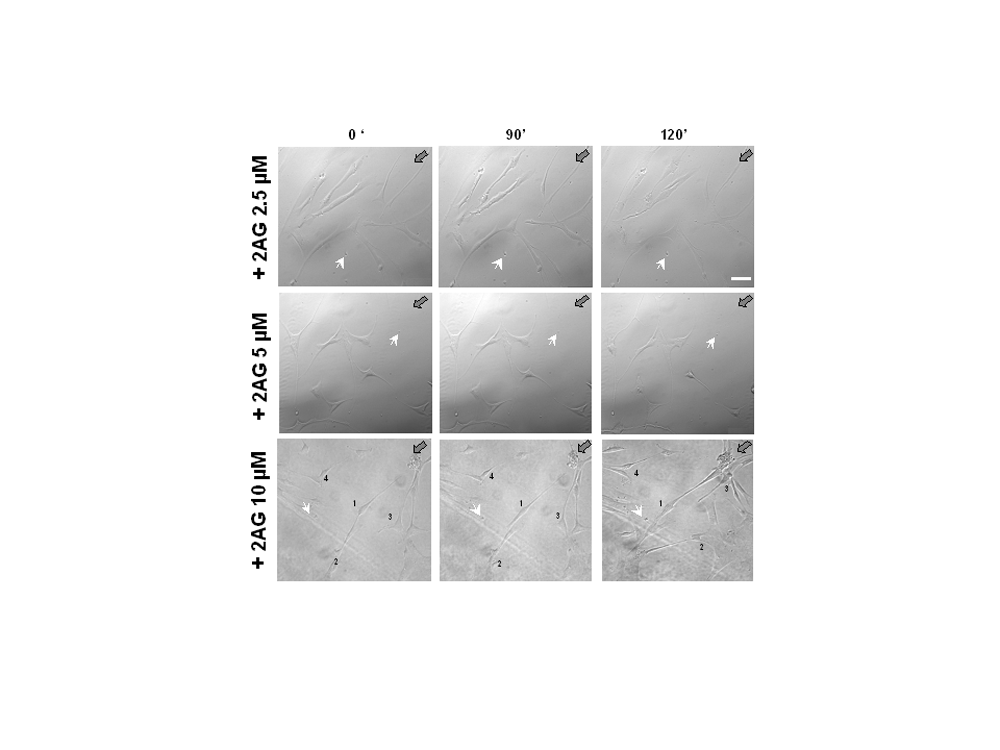

Supplement: Figure S2 — 2-AG chemoattractant properties. Panel shows the migratory effect induced by three different concentrations of 2-AG, [2.5 µM], [5 µM] and [10 µM], respectively, recorded at starting point (0 min), 90 min and 120 min from the 2-AG (filled gray arrows) or vehicle (white arrows) exposure. (TIF) [file pone.0080022.s002.tif]
